# Medicare Advantage Civil Monetary Penalties and Profits

**DOI:** 10.1001/jamahealthforum.2026.0217

**Published:** 2026-04-03

**Authors:** Jeffrey Marr, Zihan Chen, Kendra Offiaeli, David J. Meyers

**Affiliations:** 1 Department of Health Services, Policy and Practice, Brown University School of Public Health, Providence, Rhode Island

## Abstract

This cross-sectional study examines how civil monetary penalties compare to the profits of Medicare Advantage insurers to understand whether they are large enough to be a meaningful deterrent to violating program rules.

## Introduction

A majority of Medicare beneficiaries are now enrolled in Medicare Advantage (MA), where private insurers provide coverage to enrollees.^1^ The Centers for Medicare & Medicaid Services conduct oversight of MA insurers to ensure that they comply with the program’s rules and can fine MA insurers that violate these rules using civil monetary penalties. These penalties may be issued to insurers for inappropriately delaying or denying services, charging incorrect premiums or out-of-pocket costs, or for providing inaccurate explanations of plan benefits.^2^ We examined how these civil monetary penalties compare to the profits of MA insurers to understand whether they are large enough to be a meaningful deterrent to violating program rules.

## Methods

We combined data on Centers for Medicare & Medicaid Services civil monetary penalties with data on insurer finances. First, we obtained the list of civil monetary penalties from 2010 to 2024 with a Freedom of Information Act request. Second, we used data from Mark Farrah Associates (MFA) on the finances of MA insurers, which is primarily collected through National Association of Insurance Commissioners filings.

We began with 181 insurer-years that received penalties and manually matched these to insurer-years in the MFA data. We excluded 35 insurer-years that did not appear in the MFA data and 12 insurer-years that had incomplete MA profitability data. The final sample of 134 insurer-years represented 74.0% of penalized insurer-years and 97.6% of enrollment-weighted insurer-years.

Following past research, our primary measure of insurer profitability was MA gross margins, the difference between MA premiums and claims.^3^ As a secondary measure of insurer profitability, we also examined the insurers’ net income. While this measure includes health insurance market segments outside of MA, it also accounts for the insurers’ administrative expenses among other costs outside of medical claims. All values are expressed in 2024 US dollars using the Consumer Price Index.

This study followed Strengthening the Reporting of Observational Studies in Epidemiology (STROBE) reporting guidelines. In accordance with the Common Rule (45 CFR §46), we did not seek institutional review board approval because the study involved no human participants. Analysis was conducted using Stata, version 18 (StataCorp).

## Results

**Figure 1.**
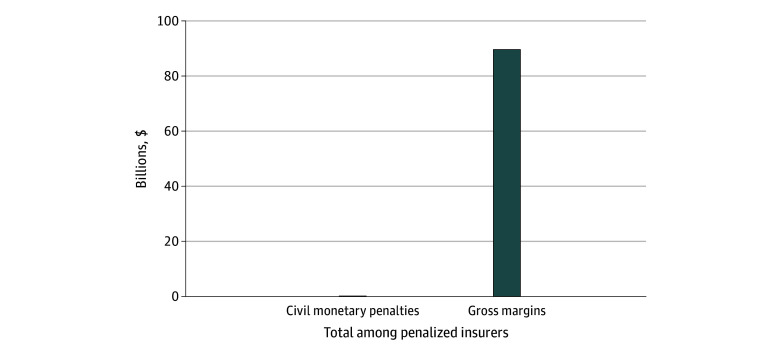
Total Civil Monetary Penalties and Gross Medicare Advantage Margins Among Penalized Medicare Advantage Insurers, 2010-2024 Data are from Mark Farrah Associates and a Freedom of Information Act request. Only insurer-years that received a civil monetary penalty were included. Gross margins are the difference between Medicare Advantage premiums and claims. Values are expressed in 2024 US dollars using the Consumer Price Index.

Between 2010 and 2024, MA insurers in the sample received $35 634 031 in civil monetary penalties (Figure 1). During the years that those insurers were penalized, they earned a total of $89 560 077 821 in gross margins. Total penalties represented 0.04% of gross margins. During those same years, penalized insurers earned $59 054 756 451 in net income across all of their lines of health insurance business.

**Figure 2.**
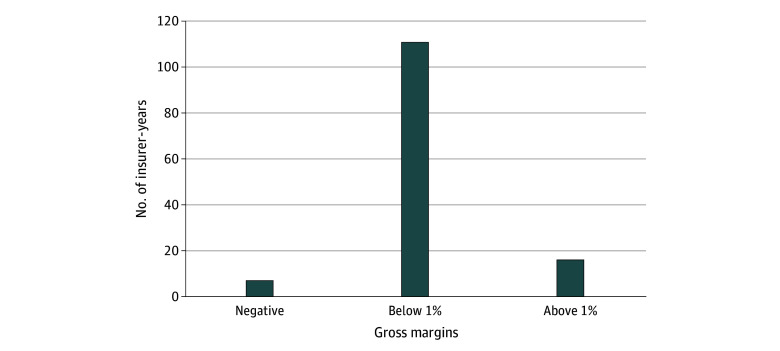
Medicare Advantage Civil Monetary Penalties as a Percentage of Gross Margins, 2010-2024 Data are from Mark Farrah Associates and a Freedom of Information Act request. Only insurer-years that received a civil monetary penalty were included.

The majority of civil monetary penalties (111 insurer-years [82.8%]) composed less than 1% of the insurer’s gross margins in the year they were penalized (Figure 2). A small number of insurer-years had negative gross margins during the year, so the penalty amount exceeded their margins (7 insurer-years [5.2%]). Another small number of insurer-years had penalties that composed more than 1% of gross margins (16 insurer-years [11.9%]).

## Discussion

In this cross-sectional study, we found that, between 2010 and 2024, civil monetary penalties issued to MA insurers were a small fraction of the money these insurers earned participating in the program. To keep from levying burdensome fines on small plans, per-enrollee civil monetary penalties are capped based on the total enrollment of the parent company. However, the cap for the largest insurers who enroll more than 3 million people is $2 million, even though these firms often earn billions of dollars in profits each year.^2,3,4^ A limitation of this analysis is that gross margins exclude plans’ administrative expenses. The small size of civil monetary penalties compared to plan profits likely makes them an ineffective deterrent of program violations in MA.

## Notes

Supplement. Data Sharing Statement
