# Supplementary material for: Medicare Advantage Civil Monetary Penalties and Profits
Source: JAMA Health Forum. 2026 Apr 3;7(4):e260217. doi: 10.1001/jamahealthforum.2026.0217 (PMC13049488; doi:10.1001/jamahealthforum.2026.0217)
Supplement: Supplement. — Data Sharing Statement [file jamahealthforum-e260217-s001.pdf]

Data Sharing Statement

Marr. Medicare Advantage Civil Monetary Penalties and Profits. *JAMA Health Forum*. Published April 03, 2026. doi:10.1001/jamahealthforum.2026.0217

Data
Data available: No
